# Generation of Col2a1-EGFP iPS Cells for Monitoring Chondrogenic Differentiation

**DOI:** 10.1371/journal.pone.0074137

**Published:** 2013-09-16

**Authors:** Taku Saito, Fumiko Yano, Daisuke Mori, Shinsuke Ohba, Hironori Hojo, Makoto Otsu, Koji Eto, Hiromitsu Nakauchi, Sakae Tanaka, Ung-il Chung, Hiroshi Kawaguchi

**Affiliations:** 1 Bone and Cartilage Regenerative Medicine, Faculty of Medicine, University of Tokyo, Bunkyo-ku, Tokyo, Japan; 2 Sensory & Motor System Medicine, Faculty of Medicine, University of Tokyo, Bunkyo-ku, Tokyo, Japan; 3 Center for Disease Biology and Integrative Medicine, Faculty of Medicine, University of Tokyo, Bunkyo-ku, Tokyo, Japan; 4 Division of Stem Cell Therapy, Center for Stem Cell Biology and Regenerative Medicine, Institute of Medical Science, University of Tokyo, Minato-ku, Tokyo, Japan; 5 Clinical Application Department, Center for iPS Research and Application (CiRA), Kyoto University, Shogoin, Sakyo-ku, Kyoto, Japan; University of Minnesota, United States of America

## Abstract

Induced pluripotent stem cells (iPSC) are a promising cell source for cartilage regenerative medicine; however, the methods for chondrocyte induction from iPSC are currently developing and not yet sufficient for clinical application. Here, we report the establishment of a fluorescent indicator system for monitoring chondrogenic differentiation from iPSC to simplify screening for effective factors that induce chondrocytes from iPSC. We generated iPSC from embryonic fibroblasts of *Col2a1-EGFP* transgenic mice by retroviral transduction of *Oct4*, *Sox2*, *Klf4*, and *c-Myc*. Among the 30 clones of *Col2a1-EGFP* iPSC we established, two clones showed high expression levels of embryonic stem cell (ESC) marker genes, similar to control ESC. A teratoma formation assay showed that the two clones were pluripotent and differentiated into cell types from all three germ layers. The fluorescent signal was observed during chondrogenic differentiation of the two clones concomitant with the increase in chondrocyte marker expression. In conclusion, *Col2a1-EGFP* iPSC are useful for monitoring chondrogenic differentiation and will contribute to research in cartilage regenerative medicine.

## Introduction

Osteoarthritis (OA) is the most common joint disorder and is characterized by cartilage degradation [1], [2]. Because articular cartilage has a poor regenerative capacity, regeneration using tissue engineering methods may be a greatly beneficial and innovative medical treatment for OA [3]. Many studies have focused mainly on the use of articular chondrocytes or somatic stem cells as a potent cell source for cartilage regenerative medicine [4]. Although these types of cells are useful for repairing focal cartilage defects, application of these cells for repairing massively degraded cartilage in OA joints is difficult because of their limited proliferative potential in vitro [5], [6]. Recently, induced pluripotent stem cells (iPSC) have been considered a promising cell source for regenerative medicine because of their pluripotency and proliferative activity. However, an efficient method of chondrocyte induction from iPSC that is sufficient for clinical application has not been established.

Type II collagen (Col2a1) is the principal and specific matrix protein in cartilage that is produced by chondrocytes, and the promoter/enhancer of *Col2a1* has been widely used to screen for chondrogenic differentiation [7], [8]. Previously, we established an indicator ATDC5 cell line for chondrogenic differentiation using four repeats of a *COL2A1* enhancer ligated to a basal promoter and fluorescent reporter. Using this system, we identified Sorting Nexin 19 as a novel chondrogenic factor and a new thienoindazole derivative (TD-198946) as a potent chondrogenic small compound [9], [10].

Here, we describe the generation of iPSC from embryonic fibroblasts of *Col2a1-EGFP* transgenic mice to simplify screening for effective factors that induce chondrocytes from iPSC. In the present study, we confirmed the pluripotency of the generated iPSC and validated the fluorescence following stimulation with insulin, bone morphogenetic protein 2 (BMP-2), transforming growth factor beta 1 (TGF-β1), a Notch signaling ligand, a Wnt signaling activator, and TD-198946.

## Materials and Methods

### Generation of iPSC

We generated iPSC using concentrated vesicular stomatitis virus-G-retroviral supernatant as described previously [11], [12]. 293GPG cells were a kind gift from Dr. R.C. Mulligan (Children’s Hospital Boston, Harvard Medical School) [13].

### Cell Culture

We purchased mouse embryonic stem cells (ESC) from Riken BRC. We isolated mouse embryonic fibroblasts (MEF) as described previously [14]. We cultured ESC and iPSC with mitomycin C-inactivated MEF as feeder cells in ES medium (Knockout DMEM [Gibco] supplemented with 15% knockout serum replacement [KSR; Gibco], 2 mM l-glutamine [Gibco], 1% [vol/vol] nonessential amino acids [Gibco], 0.1 mM 2-mercaptoethanol [Sigma], 50 units/mL penicillin [Sigma], 50 µg/mL streptomycin [Sigma]) with 1,000 units/mL leukemia inhibitory factor (Millipore). For differentiation, we induced formation of embryoid bodies (EBs) from iPSC as previously described [15] and cultured them in suspension on petri dishes for 5 days in ES medium with 10 nM retinoic acid (Sigma). We then individually plated the EBs onto gelatin-coated 24-well plates and cultured them for an additional 12 days in basal differentiation medium (DMEM/F12 [Gibco] supplemented with 2 mM l-glutamine, 1% [vol/vol] nonessential amino acids, 2% [vol/vol] B27 [Gibco], and 0.1 mM 2-mercaptoethanol) with or without each supplement. We tested 1% (vol/vol) insulin/transferrin/selenium (ITS) supplement (Gibco), 10 ng/mL BMP-2 (Peprotech), 10 ng/mL TGF-β1 (Peprotech), 10 µM Jag-1 peptide (StemRD; Notch ligand), 3 µM CHIR99021 (Merck Millipore; glycogen synthase kinase 3 beta [GSK-3β] inhibitor), and 10 nM TD-198946. For high cell-density micromass culture, we gently dissociated 5-day-old EBs with 0.25% (vol/vol) trypsin for 3 min at 37°C, spotted 20-µL drops containing 5 × 10^5^ cells onto gelatin-coated 12-well plates, and cultured them for an additional 12 days in basal differentiation medium with or without each supplement. After differentiation, we stained the cells with 0.3% Alcian blue 8 GX (Sigma) in 3% acetic acid.

### Teratoma Formation and Histological Analysis

We injected 1 × 10^6^ iPSC into the testes of male severe combined immunodeficient (SCID) mice, as previously described [12]. Eight weeks after injection, the mice were sacrificed, and the resultant tumors were dissected, fixed in 4% paraformaldehyde, embedded in paraffin, sectioned, and stained with hematoxylin and eosin.

### Real-time RT-PCR

We isolated total RNA with an RNeasy mini kit (Qiagen) and reverse transcribed 1 µg total RNA with MultiScribe reverse transcriptase (Applied Biosystems). We performed real-time RT-PCR with an Mx3000P (Agilent) apparatus. Each PCR reaction contained 1× FastStart Universal SYBR Green Master (Roche), 0.3 µM specific primers, and 20 ng cDNA. We calculated the mRNA copy number of each specific gene in total RNA using a standard curve generated by serially diluted plasmids containing PCR amplicon sequences. We normalized the copy number to rodent total RNA (Applied Biosystems) with mouse glyceraldehyde-3-phosphate dehydrogenase (*Gapdh*) as an internal control. We ran all reactions in triplicate. Primer sequence information is available upon request.

### Animals

We performed all experiments according to a protocol approved by the Animal Care and Use Committee of the University of Tokyo.

## Results

**Figure 1:**
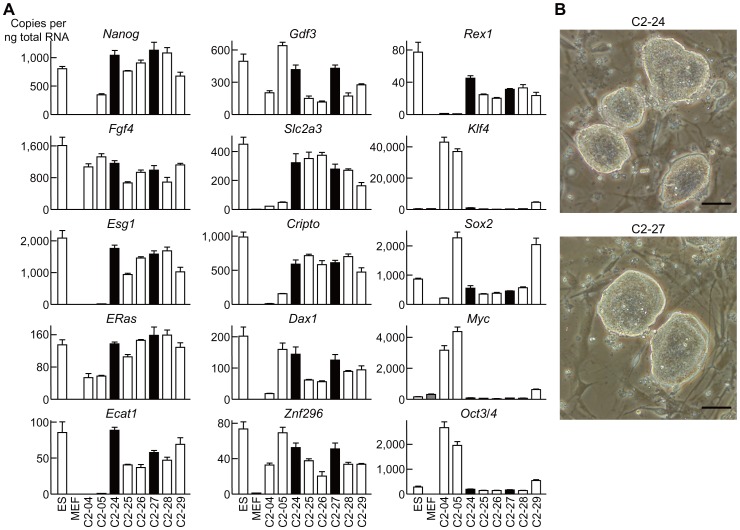
Generation of iPSC from MEF of *Col2a1-EGFP* transgenic mice. (A) mRNA levels of ESC marker genes and iPSC factors in ESC, MEF, and generated iPSC clones. Data are expressed as means (bars) ± SDs (error bars) for four wells/group. (B) Morphology of clones C2-24 and C2-27. Scale bars, 50 µm.

We prepared MEF from *Col2a1-EGFP* transgenic mouse [16] embryos (E12.5) and transduced the MEF with a cocktail of retroviral vectors harboring each of the iPSC factor genes *Klf4*, *Sox2*, *Oct4*, and *c-Myc*. We picked up iPSC-like colonies 10 days after transduction, expanded them, and analyzed mRNA expression levels of ESC marker genes and iPSC factor genes. The results of gene expression in representative clones among the 30 established clones (C2-01 through 30) are shown in Figure 1A. Expression patterns of ESC marker genes in clones C2-24 and C2-27 were most similar to those of the ESC. Moreover, expression of the four transduced iPSC factors was silenced to a level similar to in the ESC (Figure 1A). These two clones were also morphologically similar to ESC (Figure 1B).

**Figure 2:**
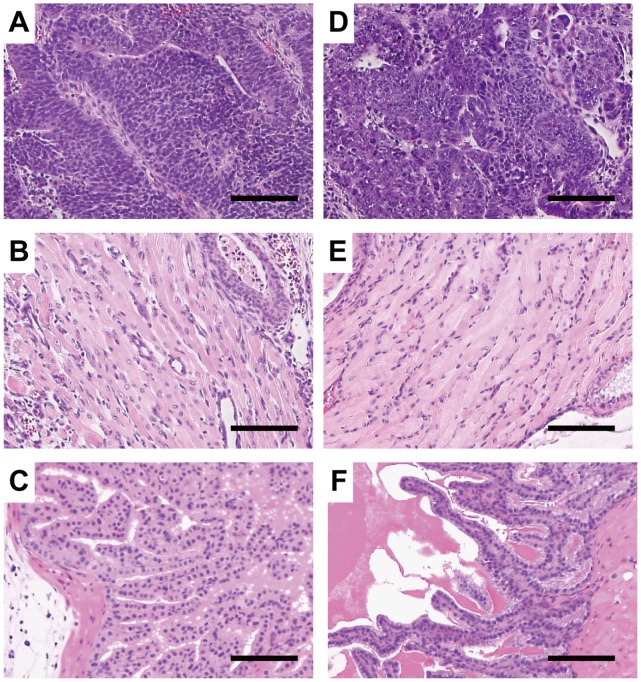
*In vivo* differentiation of C2-24 and C2-27 with the teratoma formation assay. Various tissue types are present in teratomas derived from C2-24 (A–C) and C2-27 (D–F). (A, D) Neural tissues. (B, E) Muscle. (C, F) Intestinal epithelia. Scale bars, 100 µm.

Next, we performed teratoma formation assays in the testes of SCID mice to examine the pluripotency of C2-24 and C2-27. Eight weeks after injection, histological analysis demonstrated that the teratomas derived from the two clones included various types of tissues. Neural tissues (ectoderm), muscle (mesoderm), and intestinal epithelia (endoderm) were histologically identified in both C2-24- and C2-27-derived teratomas (Figure 2). These results suggested that C2-24 and C2-27 were pluripotent and could differentiate into cell types of all three germ layers.

**Figure 3:**
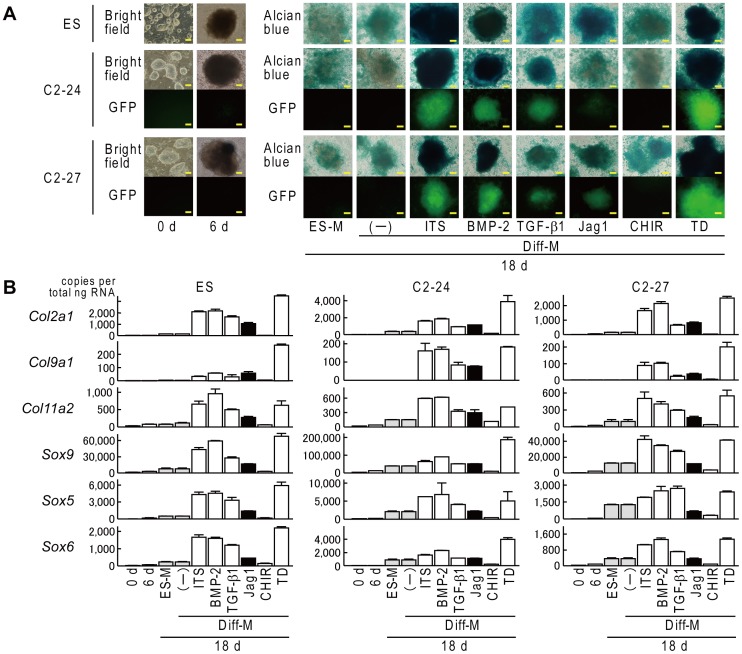
Fluorescence during chondrogenic differentiation of ES, C2-24, and C2-27 via EB formation. (A) Morphology, Alcian blue staining, and fluorescent view. 0 d: during maintenance on feeder cells, 6 d: 1 day after plating of EBs, 18 d: after an additional 12 days of culture, ES-M: ES medium, Diff-M: basal differentiation medium, Jag1: Jag-1 peptide, TD: TD-198946. Scale bars, 50 µm. (B) mRNA levels of chondrocyte-specific collagen genes and Sox genes. Data are expressed as means (bars) ± SDs (error bars) for four wells/group.

We then validated the fluorescence intensity of C2-24 and C2-27 during chondrogenic differentiation. We used mouse ESC (Riken BRC) as a control clone for differentiation. Fluorescence was not detected in either iPSC clone during maintenance culture on feeder cells (0 d) or 1 day after plating of EBs (6 d) (Figure 3A). When we differentiated the EBs in differentiation medium for an additional 10 days, an intense GFP signal was detected in both iPSC clones differentiated with ITS, BMP-2, or TD-198946, and a weak GFP signal was detected in iPSC clones differentiated with TGF-β1 or the Jag-1 peptide; however, the signal was barely detected when clones were cultured in ES medium, differentiated with no supplement, or differentiated with CHIR99021 (Figure 3A). We further confirmed the chondrogenic differentiation of these clones with Alcian blue staining and quantitation of mRNA for chondrocyte-specific collagen genes and Sox genes (Figure 3A, 3B). Alcian blue staining of the differentiated EBs was comparable with the GFP signal in both clones (Figure 3A). The mRNA levels of *Col2a1* as well as other collagen genes and Sox genes were strongly increased when differentiated with ITS, BMP-2, or TD-198946, confirming that the g003fluorescence signal was induced parallel to the endogenous expression of Col2a1 (Figure 3B).

**Figure 4:**
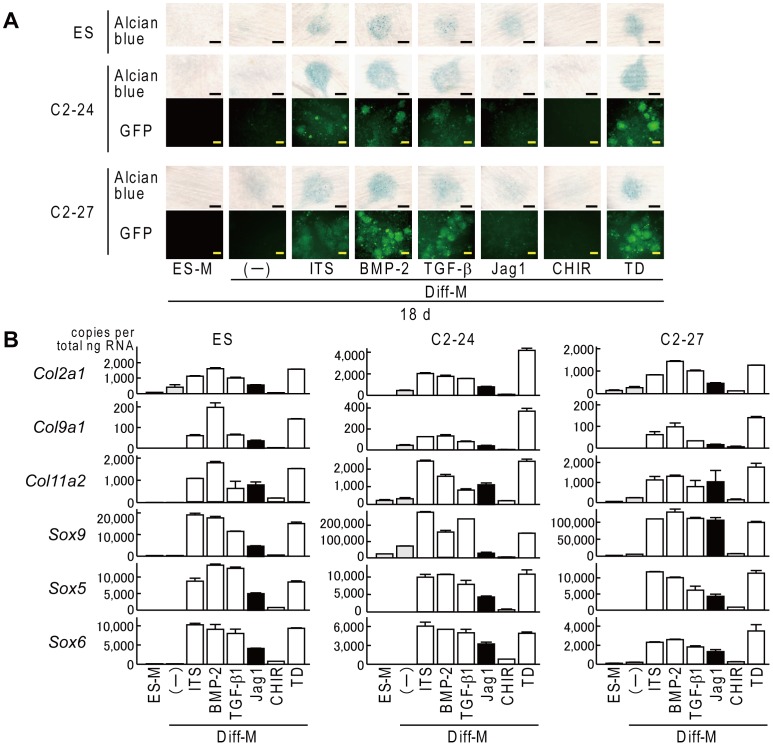
Fluorescence during chondrogenic differentiation of ES, C2-24, and C2-27 in high cell-density micromass culture. (A) Alcian blue staining and fluorescent view. ES-M: ES medium, Diff-M: basal differentiation medium, Jag1: Jag-1 peptide, TD: TD-198946. Scale bars, 20 mm for Alcian blue staining and 50 µm for fluorescent view. (B) mRNA levels of chondrocyte-specific collagen genes and Sox genes. Data are expressed as means (bars) ± SDs (error bars) for four wells/group.

Finally, we validated the fluorescence intensity of both clones when differentiated in high cell-density micromass culture. An intense GFP signal was detected in both iPSC clones differentiated with ITS, BMP-2, TGF-β1, or TD-198946, whereas a weak signal was detected when clones were cultured with the Jag-1 peptide or CHIR99021 (Figure 4A). Alcian blue staining and the mRNA levels of collagen genes and Sox genes were comparable with the GFP signal in both clones (Figure 4B).

## Discussion

Generation, differentiation, and maturation of chondrocytes *in vivo* are regulated by many factors and signals including fibroblast growth factor, parathyroid hormone-related peptide, Indian hedgehog, BMPs, WNT, and Notch[17]–[20]. Many of these factors and signals are useful for chondrocyte induction *in vitro.* Furthermore, small compounds that induce chondrocytes from stem cells or progenitor cells have been reported recently [10], [21]. However, an efficient method for inducing chondrocytes from iPSC for clinical application has not been established. For practical use of iPSC in cartilage regenerative medicine, culture conditions including various types of cytokines and compounds should be optimized. The *Col2a1*-*EGFP* iPSC developed in the present study will permit real-time monitoring of chondrogenic differentiation, will simplify the process of optimization, and will contribute to establishing a better protocol for chondrocyte induction.

The present study confirmed that TD-198946 is a chondrogenic factor as potent as insulin, BMP-2, and TGF-β1. TD-198946 has two excellent effects: induction of chondrogenic differentiation and suppression of chondrocyte hypertrophy [10]. Chondrogenic factors including insulin, BMP-2, and TGF-β1 enhance chondrocyte hypertrophy as well as chondrogenic differentiation [10], [22], [23]; however, chondrocyte hypertrophy is undesirable for cartilage regenerative medicine because it leads to degeneration of cartilage and bone formation. A combination of these potent factors including TD-198946 may provide a better protocol for inducing chondrocytes that are suitable for cartilage regenerative medicine from iPSC. We also confirmed that the activation of Notch signaling or Wnt signaling did not enhance the chondrogenic differentiation of iPSC. Inhibition of these signaling pathways may be useful for refining differentiation, because both signaling pathways can suppress chondrogenic differentiation[20], [24]–[26].

EB formation is one of the most efficient methods for differentiating ESC or iPSC in early stages. However, EB formation is not suitable for mass tissue production because of limited cell proliferation. When we differentiated the C2-24 and C2-27 clones in a monolayer culture using the same factors described above, neither a significant increase in Col2a1 mRNA nor a fluorescent signal was observed with real-time RT-PCR or flow cytometry (data not shown), indicating that a combination of factors including other compounds may be necessary to induce chondrocytes efficiently from iPSC in monolayer culture.

In the present study, we generated iPSC from *Col2a1-EGFP* transgenic mice. We also transduced an exogenous reporter construct into ATDC5 cells for screening of chondrogenic differentiation. Recently, Diekman et al. used the exogenous reporter construct *Col2a1-EGFP* to detect chondrogenic differentiation of mouse iPSC [27]. Although the exogenous transduction of the reporter construct may impair the properties of the cells by integrating into genomic DNA, this procedure can be used in human cells, especially in human iPSC. These reporter systems are beneficial for developing better chondrogenic differentiation methods from iPSC.
